
JOURNAL INFORMATION
==============================
NLM Title Abbreviation: Wirtschaftsdienst
Journal ID: Wirtschaftsdienst
NLM Journal ID: 101086612

Wirtschaftsdienst (Hamburg, Germany : 1949)

ISSN: 0043-6275

ARTICLE INFORMATION
==============================
PMCID: PMC7765691
PMID: 33390620
DOI: 10.1007/s10273-020-2796-y
Article ID: 2796
Article version: 1
Subjects: Zeitgespräch

Build back better: Neustart transatlantischer Handelsbeziehungen?

Mildner Stormy-Annika S.Mildner@bdi.eu
1
Allmendinger Elisabeth E.Allmendinger@bdi.eu
2
1 grid.469691.5 0000 0001 0465 2215 Außenwirtschaftspolitik, BDI e.V., Breite Straße 29, 10178 Berlin, Deutschland
2 grid.469691.5 0000 0001 0465 2215 Personal- und Organisationsentwicklung, BDI e.V., Breite Straße 29, 10178 Berlin, Deutschland
Electronic publication date: 2020 Dec 26
Print publication date: 2020
Volume: 100
Issue: 12
First page: 913
Last page: 918
Copyright: © Der/die Autor(en) 2020
License: Dieser Artikel wird unter der Creative Commons Namensnennung 4.0 International Lizenz (https://creativecommons.org/licenses/by/4.0/deed.de) veröffentlicht.
Open Access wird durch die ZBW — Leibniz-Informationszentrum Wirtschaft gefördert.
License URL: https://creativecommons.org/licenses/by/4.0/

issue-copyright-statement: © The Author(s) 2020

==============================
Dr. Stormy-Annika Mildner leitet die Abteilung Außenwirtschaftspolitik des Bundesverbands der deutschen Industrie (BDI) und ist Adjunct Lecturer an der Hertie School of Governance in Berlin.

„Build back better“ – unter dieses Motto setzt Joe Biden seine anstehende Präsidentschaft. Seine Wahl zum 46. Präsidenten der Vereinigten Staaten fällt in die Zeit einer globalen Pandemie mit dramatischen und noch nicht absehbaren Konsequenzen für die Gesundheit von US-Bürger*innen, für den gesellschaftlichen Zusammenhalt und für die wirtschaftliche Lage der USA. Biden setzt auf den Wiederaufbau des Landes. Er hat sich vorgenommen, eine stark polarisierte US-Gesellschaft zu versöhnen und die internationale Reputation der USA wieder zu stärken.

Sowohl politisch als auch gesellschaftlich sind die USA heute tiefer gespalten als noch vor der Amtsübernahme Donald Trumps. So wies nicht nur der Kongress in seinem Abstimmungsverhalten während der vergangenen vier Jahre die stärkste jemals gemessene Polarisierung auf und blockierte somit viele wichtige Gesetzesvorhaben (Quirk et al., 2019), sondern auch gesellschaftlich zeigen sich tiefe Risse. Meinungsumfragen zu politischen Kernthemen wie Chancengleichheit, Immigration und Religion zeigen, wie sich die gesellschaftliche Spaltung entlang der Parteilinien von Demokraten und Republikanern während der letzten vier Jahre verstärkt hat (Pew, 2020a).

Joe Biden steht vor der Herausforderung, eine Politik zu finden, die das Land eint und von einem Großteil der Gesellschaft – und dabei nicht nur von Bidens eigenen Wähler*innen – mitgetragen wird. Die US-Wahlen haben gezeigt, dass Trump mehr Rückhalt in der Bevölkerung hat als viele im Voraus für möglich gehalten hätten. Im Vergleich zu 2016 konnte Trump sogar noch mehr Wähler*innen für sich mobilisieren und seinen Unterstützerkreis um 17,2 % vergrößern (CNN, 2016 und 2020). Betrachtet man die heiß umkämpften Bundesstaaten des Rust Belt, die Trump in der vorherigen Wahl zum Sieg verhalfen, so erhielt Trump auch dort viel Unterstützung und konnte Indiana, Iowa, Ohio – teilweise sogar mit deutlichem Vorsprung – wieder für sich gewinnen.

Joe Biden wird in den kommenden Monaten auf parteiübergreifende Zusammenarbeit angewiesen sein. Neben den von ihm angestrebten Steuererhöhungen steht auch die Verabschiedung eines Corona-Konjunkturpakets an, wofür er Unterstützung beider politischer Lager brauchen wird. Für den Beginn seiner Präsidentschaft setzt sich Biden vier Prioritäten: Die Bewältigung der Corona-Pandemie, die wirtschaftliche Erholung, der Einsatz gegen strukturellen Rassismus und der Kampf gegen den Klimawandel.

Anders als sein Vorgänger, der in seinem Programm Make America Great Again vor allem auf unilaterale Maßnahmen setzte, nahm Biden schon wenige Tage nach den Wahlen den Kontakt zu ausländischen Staatsoberhäuptern auf. Ende November 2020 verständigte er sich mit EU-Kommissionspräsidentin Ursula von der Leyen darauf, den transatlantischen Beziehungen einen neuen Impuls zu geben. Biden sieht die Stärke der USA in engen Beziehungen zu den traditionellen Verbündeten. Im Vergleich zu Trump ist Biden von internationaler Rechtsprechung sowie von der Bedeutung internationaler Organisationen überzeugt. Sein Regierungsstil verspricht, deutlich berechenbarer und kooperativer zu sein. Biden bringt viel Erfahrung mit. Er ist ein ausgewiesener Europakenner und Transatlantiker. Nachdem die transatlantischen Beziehungen während der vergangenen vier Jahre stark belastet waren, knüpfen viele in Europa große Hoffnungen an einen Neustart in der transatlantischen Zusammenarbeit.

Dies wird jedoch kein Selbstläufer sein, sondern viel Arbeit auf beiden Seiten des Atlantiks erfordern. So gehört Handel nicht zu den Top-Prioritäten von Biden. Eine proaktive Handelspolitik würde aufgrund der innenpolitischen Lage dem Präsidenten den Einsatz von viel politischem Kapital abverlangen. Zudem werden sich bestimmte transatlantische Konfliktthemen, wie beispielsweise die US-Russlandsanktionen, nicht einfach in Luft auflösen. Umso erfreulicher ist, dass die Europäische Kommission bereits einen Vorschlag für eine positive Agenda vorgelegt hat.

Verfassungsrechtlich liegt die Verantwortung für die Handelspolitik beim Kongress, wenngleich dieser die Kompetenzen der Exekutive wiederholt erweitert hat. Beispiele hierfür sind Abschnitt 232 des Handelsgesetzes von 1962 (Zölle zum Schutz der nationalen Sicherheit) oder auch Abschnitt 301 des Handelsgesetzes von 1974 (Maßnahmen gegen unfaire Handelspraktiken im Ausland) – zwei Instrumente, die Präsident Trump wiederholt nutzte. Biden wird jedoch keine Handelspolitik am Kongress vorbei machen können und wollen. Wie also steht der Kongress zum Handel? Und wie ist das Stimmungsbild in der Bevölkerung?

## Bidens innenpolitische Handlungsspielräume

Dem Umfrageinstitut Gallup zufolge ist die Einstellung der US-Bevölkerung zum Handel so positiv wie seit 25 Jahren nicht mehr (Saad, 2019). Dies gilt sowohl für demokratisch als auch republikanisch wählende Befragte. Während sich die Zustimmungsrate bereits seit 2011 deutlich verbessert hatte, sprang sie nach Amtsantritt Trumps noch einmal merkbar an. Im Februar 2019 waren laut Gallup-Umfrage fast drei von vier befragten US-Amerikaner*innen (74 %) der Meinung, dass der Handel „eine Chance für wirtschaftliches Wachstum durch US-Exporte“ darstellt (Saad, 2019). Weniger als 21 % sahen im Handel eher „eine Bedrohung für die Wirtschaft durch ausländische Importe“. Etwas weniger positiv fällt das Ergebnis aus, wenn nach Handelsabkommen gefragt wird. Einer Umfrage des Meinungsforschungsinstituts Pew zufolge (2018) bewerteten 56 % der US-Bevölkerung Freihandelsabkommen positiv, unter den demokratisch Gesinnten waren es 67 %. Diese große Unterstützung unter demokratisch Wählenden ist historisch überraschend, hatten die Demokraten doch traditionell eine zögerliche Haltung gegenüber Freihandelsabkommen. Die Erklärung liegt zumindest zum Teil in einer generellen Ablehnung von Trump (Stokes, 2018).

Gleichzeitig ist die Mehrheit der US-Amerikaner*innen laut einer Gallup-Umfrage von 2017 jedoch auch der Meinung, dass die „Förderung einer günstigen Handelspolitik für die USA auf ausländischen Märkten“ ein „sehr wichtiges“ Ziel der US-Außenpolitik sei (Saad, 2019). Hinzu kommt: Laut einer Pew-Umfrage von 2018 glauben nur 36 % der Befragten, dass Handel Arbeitsplätze schafft; nur 31 % erwarten, dass Handel Lohnsteigerungen mit sich bringt (Stokes, 2018). Trumps Slogan Make America Great Again fand viel Widerhall – nicht nur unter seinen eigenen Wähler*innen. Immerhin befürworteten laut einer Pew-Umfrage 37 % steigende Stahl- und Aluminiumzölle (45 % waren dagegen) (Pew, 2018), unter den republikanisch Wählenden waren es sogar 58 % (Pew, 2018). Insbesondere in ländlichen Regionen konnte Trump mit seiner Handelspolitik punkten, obwohl die von ihm angezettelten Handelskonflikte und steigenden Zölle gerade der US-Landwirtschaft schadeten. Die Einstellungen gegenüber China haben sich in den vergangenen Jahren – also bereits vor der Corona-Pandemie – parteiübergreifend verschlechtert. Hatten 2017 noch 47 % der Befragten eine negative Einstellung gegenüber China (Pew, 2020b), waren es im Oktober 2020 73 % (Silver et al., 2020).

Beim Thema Handel kann Biden nicht auf die volle Unterstützung durch seine Partei zählen. Insbesondere der progressive, linke Flügel fürchtet den Verlust heimischer Arbeitsplätze und positioniert sich – unterstützt von den Gewerkschaften – deutlich gegen neue Handelsabkommen (Swanson, 2020). Viele Demokraten im Kongress befürworteten Trumps Zollpolitik. Auch in den Reihen der Republikaner, die traditionell Handelsabkommen positiver gegenüberstehen, fand der scheidende Präsident viel Zuspruch. Dies zeigt sich auch daran, dass alle Versuche im Kongress, den Spielraum des Präsidenten bei der Verhängung von 232-Zöllen einzuschränken, an fehlenden Mehrheiten scheiterten. Besonders viel Unterstützung erhielt Trumps Chinapolitik. So solidarisierten sich führende Demokraten im Kongress mit Trumps Chinapolitik, auch wenn sie seinen politischen Stil und die Twitter-Politik kritisierten.

Der innenpolitische Handlungsspielraum Bidens wird auch durch die Mehrheitsverhältnisse im Kongress bestimmt sein. Nach jetzigem Stand erscheint eine knappe republikanische Mehrheit im Senat wahrscheinlich. Im Repräsentantenhaus behalten die Demokraten zwar ihre Mehrheit, haben in dieser Wahl jedoch einige Sitze verloren. Ein gespaltener Kongress wird Biden die Umsetzung handelspolitischer Vorhaben weiter erschweren.

## Die handelspolitische Agenda von Joe Biden

Angesichts der öffentlichen Meinung, des Stimmungsbildes unter den Demokraten und der Mehrheitsverhältnisse im Kongress ist zu erwarten, dass die USA unter Biden keine rein liberale Handelspolitik verfolgen werden. Vielmehr dürfte diese von protektionistischen Elementen durchzogen sein. Anders als der scheidende Präsident Trump dürfte Biden jedoch kooperativer vorgehen und deutliche Akzente für einen regelbasierten und multilateral orientierten neuen Ansatz setzen. Dieser neue handelspolitische Ansatz wird somit in starkem Kontrast zu Trumps Drohpolitik stehen, die Unsicherheit schaffte und der internationalen Reputation der USA schadete.

- *Zollpolitik*: Biden hat zwar angekündigt, bestehende Zölle einer kritischen Prüfung zu unterwerfen – die sofortige Abschaffung der Trump-Zölle hat er jedoch nicht versprochen. Wo für den Aufbau der heimischen Wirtschaft erforderlich, wird auch Biden vor Zöllen nicht zurückschrecken und kann sich dabei einer breiten Unterstützung in der Bevölkerung versichert sein. Anders als Trump wolle er deren Nutzen jedoch genau prüfen und sie nicht einsetzen, „um Härte vorzutäuschen“ (Washington Trade Daily, 2020). Die Hoffnungen Europas, dass Joe Biden nach Amtsantritt die 232-Zölle auf Stahl und Aluminium zügig abschafft, könnten somit leicht enttäuscht werden. Die Androhung von Zöllen auf Fahrzeuge und Fahrzeugteile dürfte hingegen erst einmal vom Tisch sein. Weder unter Republikanern noch Demokraten fanden Trumps Autozölle viel Unterstützung.

- *Reshoring und Buy American*: Biden setzt auf innenpolitische und binnenwirtschaftliche Themen. Die Förderung inländischer Produktion („Buy American“) und die Stärkung der US-amerikanischen Mittelschicht haben Priorität. US-Firmen sollen mit Strafsteuern davon abgehalten werden, Arbeitsplätze ins Ausland zu verlagern. Auch Staatsaufträge sollen prioritär an US-Unternehmen gehen. Für Unternehmen, die in den USA investieren, soll es dagegen Steuererleichterungen geben. Biden plant außerdem größere Investitionen für die Herstellung von Elektrofahrzeugen und die entsprechende Infrastruktur, um mehr als 1 Mio. Arbeitsplätze in der heimischen Automobilindustrie zu schaffen. Insbesondere bei kritischen Gütern wie Pharmazeutika und medizinischer Schutzausrüstung setzt er auf mehr Unabhängigkeit von globalen Lieferketten. Präsident Trump hatte wiederholt angekündigt, die Mitgliedschaft der USA im Übereinkommen über das öffentliche Beschaffungswesen der Welthandelsorganisation (WTO) aufkündigen zu wollen. Unter Biden werden die USA sicherlich Mitglied des Abkommens bleiben. Ein Austritt würde nicht nur die bereits angeschlagene WTO weiter unterminieren. Die USA würden zudem den Zugang zu den Beschaffungsmärkten wichtiger Handelspartner verlieren.

- *Harte Kante gegen China*: Biden dürfte den Konfrontationskurs gegenüber China in der zukünftigen US-Handelspolitik fortsetzen. Dies gilt jedoch anders als bei Trump nicht nur für die Handelspolitik, sondern auch für Menschenrechtsfragen. So wird sich der Druck auf Peking in Bezug auf Hongkong, Taiwan, Xinjian und das Südchinesische Meer erhöhen. Auch wird Biden seine Chinapolitik strategisch einbetten. Mit der Unterzeichnung des Freihandelsabkommens RCEP durch China und 14 weitere Staaten aus dem asiatisch-pazifischen Raum wurde die weltweit größte Freihandelszone geschaffen. Seit der Entscheidung Trumps 2017, die Transpazifische Partnerschaft (TPP) aufzukündigen, hat sich der Einfluss der USA in der Asien-Pazifik-Region zunehmend verringert. Joe Biden wird diesen schwindenden Einfluss nicht einfach hinnehmen. Um den Druck auf China hinsichtlich politischer und wirtschaftlicher Reformen zu erhöhen, wird Biden versuchen, seine „Verbündeten des Westens“ zu einen, um gemeinsam gegen illegale Handelspraktiken Chinas vorzugehen. Gleichzeitig dürfte er die Umsetzung des sogenannten Phase One Deals durch China weiter einfordern. In diesem hatte sich Peking verpflichtet, zusätzliche US-Produkte und Dienstleistungen zu importieren, auf erzwungenen Technologietransfer zu verzichten und seinen Markt für US-Finanzdienstleistungen stärker zu öffnen. Da der Phase One Deal viele Streitthemen noch nicht adressiert – allen voran den Umgang mit Staatsunternehmen und Subventionen – dürfte Biden auf ein zweites Abkommen mit China beharren. Im Dezember 2020 betonte Biden, keine sofortigen Schritte im Zollkonflikt unternehmen zu wollen.

- *Erst einmal kein Abschluss neuer Handelsabkommen*: Weltweit steigt die Zahl von Freihandelsabkommen. Für die USA bedeutet dies, dass sich ihr Zugang zu ausländischen Märkten relativ verschlechtert. Dennoch ist nicht damit zu rechnen, dass Biden zu Beginn seiner Präsidentschaft versuchen wird, neue Handelsabkommen zu verhandeln. Im Sommer 2021 läuft die sogenannte Trade Promotion Authority (TPA) aus. Dieses Mandat, das der Kongress dem Präsidenten gewähren kann, ist unabkömmlich für einen erfolgreichen Ratifizierungsprozess. Hat der Präsident ein Abkommen mit der TPA unterzeichnet, muss der Kongress über dieses abstimmen. Es gelten enge Fristen; inhaltlich kann der Kongress das Abkommen nicht mehr verändern. Für die Ratifizierung reichen einfache Mehrheiten in beiden Kammern. Sowohl Präsident George W. Bush als auch Präsident Barack Obama hatten die TPA nur mit knappen Mehrheiten im Repräsentantenhaus erhalten. Angesichts seiner ambitionierten innenpolitischen Agenda dürfte Biden kein politisches Kapital für die TPA opfern wollen.

- *Reform der WTO bleibt wichtig – und schwierig*: Biden zeigt sich offener für multilaterale Organisationen und Verhandlungslösungen und plant, dem Pariser Klimaabkommen wieder beizutreten. Bei der WTO sieht Biden großen Reformbedarf, erkennt aber an, diese könne unter Mitwirkung der USA ein „effektives Instrument“ sein, wie sein designierter Außenminister Tony Blinken Ende November ausführte (Bade, 2020). Darüber hinaus betont Biden die Bedeutung der westlichen Wertegemeinschaft und lässt hoffen, dass die transatlantische Zusammenarbeit intensiviert werden kann. Mit Biden öffnet sich ein Fenster, um den Konflikt um die neue WTO-Generaldirektorin beizulegen. Die USA hatten die Ernennung von Ngozi Okonjo-Iweala, die von den WTO-Mitgliedern mehrheitlich unterstützt wird, Ende Oktober blockiert. Der Konflikt um den Streitschlichtungsmechanismus dürfte sich hingegen nicht so einfach auflösen lassen. Die Kritik am Berufungsgremium, an angeblichen Mandatsüberschreitungen und der Länge der Streitschlichtungsverfahren wird überparteilich getragen. Auch unter Biden dürften die USA auf strengere Überwachungsverfahren für die Handelspolitiken der Mitglieder und Sanktionsmöglichkeiten pochen. Selbiges gilt für die besondere Behandlung von Entwicklungsländern in der WTO. Die USA fordern seit langem eine genauere Klassifizierung von Ländern nach Entwicklungsstand und die damit verbundenen Ausnahmen vom Regelwerk der WTO.

Joe Biden wird deutlich mehr als sein Vorgänger auf internationale Allianzen setzen, aber auch mehr Verantwortung von seinen Partnern erwarten. Dies wird auch das transatlantische Verhältnis prägen. Während sich die EU in ihrer globalen Verantwortung in den vergangenen vier Jahren hinter der Kritik an Trump verstecken konnte, wird Biden eine klarere geopolitische Positionierung Europas einfordern. Dies wird auch wichtige Einzelthemen wie die Frage einer Beteiligung chinesischer Firmen am Aufbau von 5G-Netzen betreffen. Auch hinsichtlich russischer Aggressionen wird ein Präsident Biden eine geschlossene europäische Ablehnung und ein klares Bekenntnis zu den USA erwarten.

Das Interesse Bidens, traditionelle Verbündete für die Umsetzung seiner außenpolitischen Ziele wieder hinter sich zu einen, sollte Europa dazu veranlassen, aktiv auf die USA zuzugehen und die Zukunft transatlantischer Zusammenarbeit mitzugestalten.

## Die transatlantische Agenda

Neben der Beilegung bestehender transatlantischer Konflikte, und dabei insbesondere der Aufhebung der Stahl- und Aluminiumzölle, muss die zukünftige transatlantische Agenda breiter gedacht werden. Statt sich allein auf handelspolitische Themen zu beschränken, müssen Möglichkeiten für erfolgreiche transatlantische Zusammenarbeit in anderen Bereichen einbezogen werden. Die transatlantische Agenda ist dann am erfolgversprechendsten, wenn Bidens innen- und binnenwirtschaftliche Prioritäten mit internationalen Themen verknüpft werden.

- *Gesundheit*: Die Corona-Pandemie hat gezeigt, wie schnell nationale Denkmuster wiederkehren können. Die Tatsache, dass zwei der erfolgreichsten Forschungsprojekte für einen COVID-19-Impfstoff unter Beteiligung US-amerikanischer und deutscher Firmen und Investoren entwickelt wurden, ist jedoch ein Positivbeispiel praktischer transatlantischer Zusammenarbeit. Die EU und die USA könnten in der Bekämpfung der Corona-Pandemie noch stärker zusammenarbeiten und sich letztendlich für einen weltweiten Zugang zu einem COVID-19-Impfstoff einsetzen. Auch im Rahmen der Weltgesundheitsorganisation besteht Hoffnung auf mehr Engagement der USA unter einer Präsidentschaft Bidens. Zudem könnten die transatlantischen Partner eng bei der Entwicklung von Bereitschafts- und Reaktionsplänen für Pandemien kooperieren. Um die Verfügbarkeit von Medikamenten und medizinischen Produkten zu verbessern, sollten sich die transatlantischen Partner gemeinsam für eine Modernisierung des plurilateralen WTO-Abkommens über den Handel mit pharmazeutischen Produkten einsetzen. Dieses beseitigt Zölle und andere Abgaben für eine große Zahl an pharmazeutischen Produkten, müsste aber dringend erweitert werden – sowohl bezüglich der Produkte als auch der Mitglieder. Anstatt wie von den USA angedroht medizinische Produkte aus dem WTO-Abkommen für das öffentliche Beschaffungswesen auszuklammern, sollten sich die USA zusammen mit der EU für die Erweiterung des Abkommens einsetzen. Es ist an der Zeit, nicht nur den Deckungsgrad auszudehnen, sondern auch die Zahl der unterstützenden Mitglieder –allen voran China – zu erhöhen.

- *Digitales*: Das Potenzial ist groß, durch transatlantische Kooperation Zukunftsthemen wie künstliche Intelligenz oder autonomes Fahren voranzubringen. Nicht nur bei Forschung und Entwicklung sollten die EU und die USA den Schulterschluss suchen, sondern auch bei der Standardsetzung. Ansonsten droht, dass neue Barrieren im transatlantischen Handel entstehen. Auch sollte der Dialog über Datenschutzstandards, die Besteuerung digitaler Dienstleistungen, Wettbewerbsrecht und Digitalplattformen verstärkt werden. In diesem Zusammenhang können auch Fragen zur Zukunft der Digitalwirtschaft geklärt werden. Dies umfasst auch die Entwicklung gemeinsamer Ansätze, wie europäische und US-amerikanische Unternehmen in ihrer Resilienz gegenüber aggressiven chinesischen Handelspraktiken gestärkt werden können. Schließlich sollte die EU einen engeren Austausch mit den USA zu den plurilateralen WTO-Verhandlungen über ein E-Commerce-Abkommen suchen.

- *Klima*: Die Ankündigung Bidens, die USA gleich zu Beginn seiner Präsidentschaft in das Pariser Klimaabkommen zurückführen zu wollen, ist ein wichtiger Schritt für mehr transatlantische Zusammenarbeit im Kampf gegen den Klimawandel. Bidens klimapolitische Ziele und der European Green Deal können, wenn sie in gegenseitiger Abstimmung umgesetzt werden, ihre Wirkung wechselseitig verstärken. Darüber hinaus sollten sich die EU und die USA über ihre energiepolitischen Ansätze austauschen. Ein potenzielles neues Minenfeld im transatlantischen Verhältnis ist der von der EU geplante $CO_2$-Grenzausgleichsmechanismus (CBAM/CBA). Ziel ist es, EU-Unternehmen wettbewerbsfähig zu halten, die sich bei verschärften Klimazielen härteren Klimaschutzanforderungen stellen müssen. Die EU sollte hierzu zügig den Dialog mit den USA suchen. Ein positiver Impuls nicht nur für die transatlantischen Beziehungen, sondern auch für das multilaterale Handelssystem würde sich aus einer Wiederbelebung der plurilateralen Verhandlungen über das sogenannte Umweltgüterabkommen ergeben. Ziel ist der Abbau von Zöllen auf Produkte, die einen positiven Effekt auf die Umwelt haben.

- *Bildung*: Die erst kürzlich unterzeichnete gemeinsame Absichtserklärung zwischen den USA und Deutschland zur Zusammenarbeit bei der Förderung dualer Ausbildung und beruflicher Bildung verdeutlicht das Potenzial enger transatlantischer Zusammenarbeit im Bereich Bildung. Der Absichtserklärung folgend sollen insbesondere qualitativ hochwertige duale Ausbildungsprogramme aufgebaut und wirksame Strategien für einen besseren Zugang zu praxisnaher Ausbildung entwickelt werden. Die Zusammenarbeit wird auf einer breiten Kooperation über verschiedene Ebenen zwischen Privatsektor, Verbänden, NGOs bis hin zu Institutionen des öffentlichen Sektors basieren.

- *Handel*: Transatlantische Zusammenarbeit im handelspolitischen Umfeld muss auf multilateraler und bilateraler Ebene erfolgen. Wichtig ist das Thema Subventionen und der Umgang mit Staatsunternehmen, zu dem bereits ein Vorschlag der Trilateralen Initiative (Japan, USA und EU) vorliegt. Nachdem es in den vergangenen Monaten sehr ruhig um die Initiative geworden ist, müsste der Faden rasch wieder aufgenommen werden. Ebenso wichtig ist das Thema Streitschlichtung in der WTO. Die EU hat mit mittlerweile 22 anderen WTO-Mitgliedern (darunter auch China) das Multi-Party Interim Appeal Arbitration Arrangement (MPIA) für die Berufung von Streitfällen aufgesetzt. Die USA sind bisher kein Mitglied. Umso wichtiger ist es, dass die EU und die USA eng bei der Reform des WTO-Streitschlichtungsmechanismus zusammenarbeiten. Auf bilateraler Ebene muss die Deeskalation bestehender Handelskonflikte an oberster Stelle stehen – allen voran der Streit über Luftfahrtsubventionen (Fall Airbus-Boeing) und die Rücknahme der Stahl- und Aluminiumzölle. Auch wenn kaum Hoffnungen auf ein umfassendes Freihandelsabkommen bestehen, so könnte Europa doch die Gespräche über ein Industriegüterabkommen sowie regulatorische Kooperationen weiter vorantreiben. Besonders wichtig ist die Zusammenarbeit bei neuen Technologien. Die EU sollte daher ihren Vorschlag eines Transatlantic Trade and Technology Council mit Nachdruck verfolgen.

Die Präsidentschaft Trumps hat die transatlantischen Beziehungen vor eine Zerreißprobe gestellt und das Selbstbewusstsein der Europäer*innen gestärkt. Die EU sollte jetzt nicht auf Angebote und Initiativen aus Washington warten, sondern ein transatlantisches Verhältnis auf Augenhöhe einfordern, eigene Prioritäten setzen und aktiv Angebote unterbreiten.


Elisabeth Allmendinger, M. A., arbeitete zur Begleitung der US-Wahlen 2020 als Trainee in der Abteilung Außenwirtschaftspolitik des BDI.


Literatur

Bade, G. (2020), Biden Won’t Rule Out New Tariffs, Adviser Says, Politico, 22. September, https://www.politico.com/news/2020/09/22/biden-tariffs-adviser-420096 (28. November 2020).
CNN (2016), Presidential Results, https://edition.cnn.com/election/2016/results (26. November 2020).
CNN (2020), Presidential Results, https://edition.cnn.com/election/2020/results/president (26. November 2020).
Pew (2018), Stark Partisan Divide on Proposed Tariff Increases on Imports of Steel and Aluminium, https://www.pewresearch.org/wp-content/uploads/2018/05/FT_18.05.10_trade-tariffs_partisan_divide.png?w=420 (27. November 2020).
Pew (2020a), Voters’ Attitudes About Race and Gender Are Even More Divided Than in 2016, https://www.pewresearch.org/poli-tics/2020/09/10/voters-attitudes-about-race-and-gender-are-even-more-divided-than-in-2016/ (21. November 2020).
Pew (2020b), Negative views of China continue to grow in U.S., https://www.pewresearch.org/global/wp-content/uploads/sites/2/2020/04/PG_2020.04.21_U.S.-Views-China_0–01.png?w=640 (27. November 2020).
Quirk, P. J., C. Lammert und M. Thunert (2019), United States Report: Sustainable Governance Indicators 2019, Bertelsmann Stiftung, https://www.sgi-network.org/docs/2019/country/SGI2019_USA.pdf (21. November 2020).
Saad, L. (2019), Americans’ Views on Trade in the Trump Era, Gallup, Oktober, https://news.gallup.com/opinion/gallup/267770/americans-views-trade-trump-era.aspx (26. November 2020).
Silver, L., K. Devlin und C. Huang (2020), Unfavorable Views of China Reach Historic Highs in Many Countries, Pew Research Center, Oktober, https://www.pewresearch.org/global/2020/10/06/unfavorable-views-of-china-reach-historic-highs-in-many-countries/ (27. November 2020).
Stokes, B. (2018), 1. Spotlight on Views of Trade in the U.S., EU and Japan, Pew Research Center, September, https://www.pewresearch.org/global/2018/09/26/spotlight-on-views-of-trade-in-the-u-s-eu-and-japan/ (26. November 2020).
Swanson, A. (2020), A Biden Win Could Renew a Democratic Split on Trade, New York Times, https://www.nytimes.com/2020/10/28/busi-ness/economy/democrats-biden-trade.html (27. November 2020).
Washington Trade Daily (2020), Biden’s Trade Policy, Washington Trade Daily, 29. Juli, 2–6.
